# Seasonal effect on fatigue, pain and dryness in primary Sjögren’s syndrome

**DOI:** 10.1186/s13075-020-2118-1

**Published:** 2020-02-24

**Authors:** Pierre-Marie Duret, Nicolas Meyer, Alain Saraux, Valérie Devauchelle-Pensec, Raphaele Seror, Véronique Le-Guern, Claire Larroche, Aleth Perdriger, Jean Sibilia, Vianney Guardiolle, Xavier Mariette, Jacques-Eric Gottenberg

**Affiliations:** 1 Department of Rheumatology, Colmar General Hospital, Colmar, France; 2 Department of Public Health, GMRC, Strasbourg University Hospital, Strasbourg, France; 3 Department of Rheumatology, Brest University Hospital, Brest, France; 4 Center for Immunology of Viral Infections and Autoimmune Diseases, Assistance Publique - Hôpitaux de Paris, Hôpitaux Universitaires Paris-Sud, Le Kremlin-Bicêtre, INSERM, Paris, France; 5 Department of Internal Medicine, Referral Center for Rare Autoimmune and Systemic Diseases, Hôpital Cochin, AP-HP, Université Paris Descartes, Paris, France; 6 Department of internal Medicine, Avicenne Hospital, Bobigny, France; 7 Department of Rheumatology, Rennes University Hospital, Rennes, France; 8 Department of Rheumatology, Referral Center for Rare Autoimmune and Systemic Diseases, Strasbourg University Hospital, Strasbourg, France; 9 CNRS, Immunopathologie et Chimie Thérapeutique/Laboratory of Excellence Medalis, Institut de Biologie Moléculaire et Cellulaire, Strasbourg, France

**Keywords:** Primary Sjögren’s syndrome, Autoimmune diseases, Seasonality, Seasons and rheumatic diseases, Outcome measures, Patient-reported outcomes (PROs), Epidemiology, Clinical trials

## Abstract

**Background:**

To assess the presence of a seasonal effect on fatigue, pain and dryness in primary Sjögren’s syndrome (pSS).

**Methods:**

Data (date; visual analogue scales (VAS) for pain, fatigue and dryness) were extracted from three randomised placebo-controlled trials (RCTs) evaluating infliximab (TRIPSS; *n* = 103 patients), hydroxychloroquine (JOQUER; *n* = 120 patients) and rituximab (TEARS; *n* = 120 patients) and from the 5-year follow-up of the ASSESS prospective cohort (*n* = 395 patients). Data were analysed at each visit for each patient, according to the day, the month of the year and the season. Linear mixed models were used to take into account the repeated structure of the data and to analyse a potential cyclic effect.

**Results:**

A total of 744, 584, 848 and 682 pain, fatigue and dryness VASs were collected on 632 subjects in spring, summer, fall and winter, respectively. No significant difference was observed in pain, fatigue and dryness, according to the month of the year or the season (all *p* values > 0.05).

**Conclusion:**

In pSS, seasonality does not affect patient-reported outcomes (PROs) on fatigue, pain and dryness.

## Background

Seasonality in rheumatic diseases is an issue frequently perceived and voiced by patients. Several studies have identified weather-related flares in rheumatoid arthritis (RA) [1, 2]. Weather conditions might also influence pain and function in osteoarthritis [3], and a seasonal pattern in gout incidence has been described [4].

In addition, cyclic seasonal variations have been associated with disease onset and activity or worsening of symptoms in immune-mediated inflammatory diseases (IMIDs), in giant cell arteritis (GCA) [5], inflammatory myopathies [6], systemic lupus erythematosus (SLE) [7] and ANCA-associated vasculitis [8]. Interestingly, seasonal variations are not associated with clinical outcomes in psoriatic arthritis [9].

However, seasonality has not been investigated in primary Sjögren’s syndrome (pSS) yet. Primary Sjögren’s syndrome is the second most frequent systemic auto-immune disease, and it is clinically characterised by the disabling triad pain, dryness and fatigue and is immunologically associated with antinuclear antibodies, anti Ro/SSA and/or La/SSB and the presence, in up to one third of the patients, of ectopic lymphoid structures developed in the target organs of the disease, which are mainly salivary and lacrimal glands [10].

Since fatigue, pain and dryness represent most of the disease burden shared by all patients with pSS, they compose the internationally validated ESSPRI (EULAR Sjögren’s Syndrome Patient Reported Index) score [11], an outcome criteria evaluated in all on-going clinical trials.

Although fatigue, pain and dryness represent a major concern for patients with pSS, there is no data available in the literature regarding the potential variations of these symptoms according to seasonality.

Based on previous reports that have demonstrated, in RA, a worsening of pain and fatigue in winter and an increase of dryness in spring in dry eye symptoms unrelated to pSS, it could be hypothesised that fall and winter are associated with increased fatigue and pain and spring and summer with exacerbation of ocular and oral dryness in pSS.

This study was therefore conducted to assess whether seasonal variations have an influence on pSS outcomes.

## Methods

### Patient selection

**Fig. 1:**
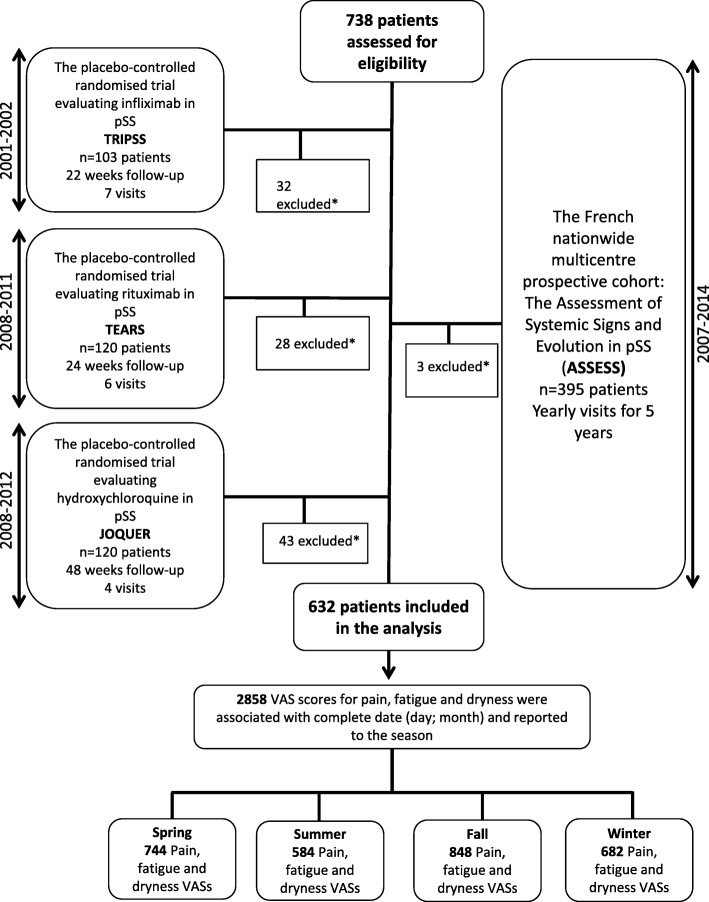
Study flow diagram. pSS, primary Sjögren’s Syndrome; VAS, visual analogue scale; *≥ 1 VAS missing data

This study analysed patients from the French nationwide multicentre pSS cohort (Assessment of Systemic Signs and Evolution in Sjögren’s Syndrome) ASSESS (*n* = 395) [12], established in 2006, with an available 5-year prospective follow-up and from three randomised placebo-controlled trials of infliximab (TRIPSS; *n* = 103; follow-up of 22 weeks; 7 visits) [13], rituximab (TEARS; *n* = 120; follow up of 24 weeks; 6 visits) [14] and hydroxychloroquine (JOQUER; *n* = 120; follow up of 48 weeks; 4 visits) [15] (Fig. 1). All patients fulfilled the American-European Consensus group criteria for pSS [16]. At each visit, in all studies, visual analogue scales (VASs) of patients for pain, fatigue and dryness were collected. Objective assessments of dryness (Schirmer test and unstimulated salivary flow) were collected in the ASSESS cohort at enrolment, second and fifth year of follow-up.

Since ASSESS is a non-interventional “natural history” cohort and all the three RCTs did not show any significant treatment effect, a combined analysis of all the available data was conducted.

### Statistical analysis

Data combined from the four previous studies were aligned according to the days, months and seasons of the year. The dataset was collected over 10 years, from 2001 to 2002 (TRIPSS), 2008 to 2011 (TEARS), 2008 to 2012 (JOQUER) and from 2007 to 2014 in the ASSESS cohort.

Patients for whom one of the three VAS criteria (fatigue, pain or dryness) was missing were not included in the analysis.

Quantitative variables are described with the mean and its standard deviation (SD). Normality was assessed using the Shapiro-Wilk test.

Mean VAS for fatigue, pain and dryness were compared first across months and, secondly, in a separate model, across seasons using linear mixed models.

Given the availability, in the ASSESS cohort, of data concerning symptomatic treatments of dryness and systemic immunomodulatory treatments (Additional file 1: Table S1), analyses were adjusted on two potential confounding factors which were age and treatment regimens assumed by patients. Linear mixed models were adjusted on symptomatic treatments of dryness (topical serum eye drops and pilocarpine hydrochloride) and systemic immunomodulatory treatments. The latter included hydroxychloroquine, corticosteroids, methotrexate, leflunomide, azathioprine, mycophenolate mofetil, cyclophosphamide (CYC) and rituximab (RTX) (CYC and RTX were considered as current treatment if prescribed in the 6 months prior to examination).

The models were fitted with a fixed time effect (month or season effect, in distinct models), with a random subject effect used to take into account the repeated structure of the data. In order to detect a cyclic seasonal effect, a cosinus transformation and a time shift was also applied to the time effect in separate models. Statistical analyses were performed using R3.3.1 with the LME4 and HGLM libraries. All statistical tests were two-sided and *p* values ≤ 0.05 were considered statistically significant.

## Results

### Variations of fatigue pain and dryness intensity between seasons

A total of 632 patients were analysed. We collected and analysed a total of 2858 VASs observations through the four studies (the number of patients withdrawn due to missing VAS data is shown on Fig. 1).

Table 1 shows the number of VASs for fatigue pain and dryness recorded in spring, summer, fall and winter and the variation of the ESSPRI score according to the season.

**Table 1:** Pain, fatigue and dryness by season for all spring, summer, fall and winter visits (*n* = 2858 observations) in 632 patients

VAS	Spring	Summer	Fall	Winter	*p* value
n*	744	584	848	682	
Pain^#^ mean (SD)	52.2 (27.9)	55.1 (28.1)	51.0 (28.7)	51.7 (28.4)	0.7541
Fatigue^#^ mean (SD)	61.9 (23.2)	62.2 (25.2)	60.0 (25.5)	61.9 (24.2)	0.7973
Dryness^#^ mean (SD)	58.9 (21.8)	61.2 (22.9)	56.9 (22.8)	57.9 (23.8)	0.4108
ESSPRI mean (SD)	57.7 (24.3)	59.5 (24.5)	55.9 (25.7)	57.2 (25.5)	0.7288

*Number of VAS; ^#^in mm; *ESSPRI* EULAR Sjögren’s Syndrome Patient Reported Index

Mean (SD) pain VAS was 52.2 (27.9) on a 100-unit scale, 55.1 (28.1), 51.0 (28.7) and 51.7 (28.4) in spring, summer, fall and winter, respectively (*p* = 0.7541). Mean (SD) fatigue was 61.9 (23.2), 62.2 (25.2), 60.0 (25.5), and 61.9 (24.2), respectively (*p* = 0.7973). Mean (SD) dryness was 58.9 (21.8), 61.2 (22.9), 56.9 (22.8) and 57.9 (23.8), respectively (*p* = 0.4108). Moreover, the ESSPRI score, which is the mean of the three VASs values for a given patient, was 57.7 (24.3), 59.5 (24.5), 55.9 (25.7) and 57.2 (25.5) on a 100-unit scale, in spring, summer, fall and winter, respectively (*p* = 0.7288). None of these fluctuations was statistically significant (*p* > 0.05).

**Fig. 2:**
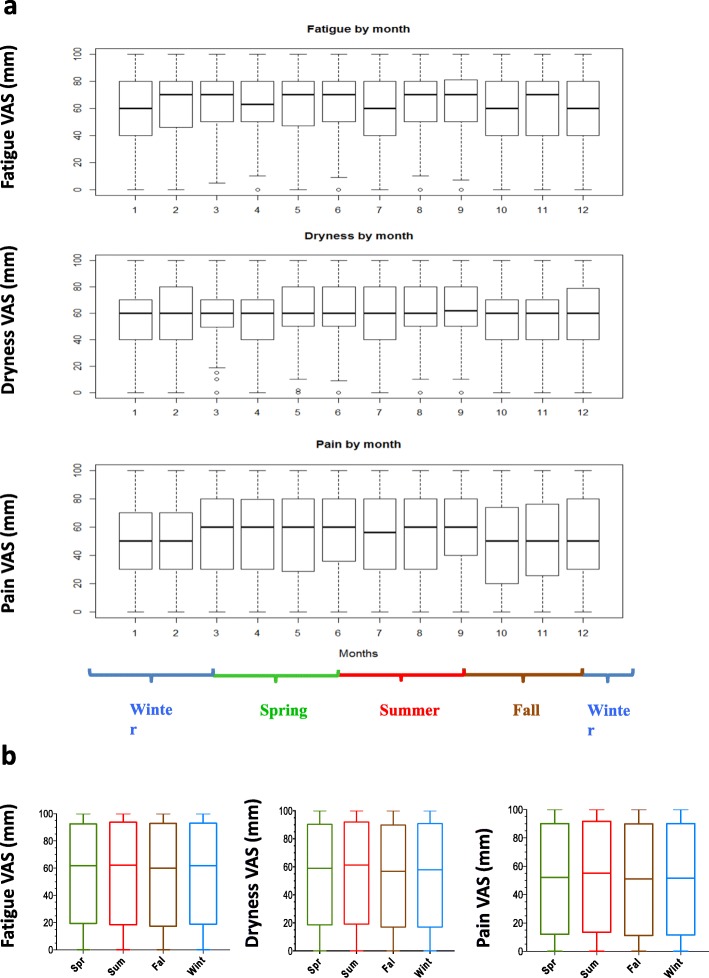
Pain, fatigue and dryness fluctuations across months and seasons. **a** Box plots of VAS variations between months from January (month: 1) to December (month: 12). **b** Box plots of VAS variations between seasons. Spring (Spr) is depicted in green, summer (Sum) in red, fall (Fal) in brown and winter (Wint) in blue

In addition, variations from month to month (Fig. 2a) or season to season (Table 1; Fig. 2b) of mean pain, fatigue and dryness were not significant either. Maximum between-months variation for pain was 7.2 on a 100-unit scale, 7.7/100 and 5.5/100 for dryness and fatigue, respectively. In addition, using cosine transform of time effect and different time lags, no time effect was observed in any model (data not shown).

Moreover, multivariate analyses were performed in the ASSESS cohort to address the influence on objective assessments of dryness (Schirmer test and unstimulated salivary flow) of potential confounding factors, such as age of patients, symptomatic treatments of dryness and systemic immunomodulatory treatments. Adjusted odds ratios (aOR) on age and treatment regimens did not detect any statistically significant seasonal effect on Schirmer test, with aOR of having a pathological Schirmer test (≤5 mm) of 0.93 CI 95% [0.42–1.8] (*p* = 0.82), 0.95 CI 95% [0.43–2.14] (*p* = 0.89) and 0.75 CI 95% [0.38–1.53] (*p* = 0.35) in spring, summer and winter, respectively, in comparison to values collected in fall. Treatment regimens did not show any influence on salivary flow either, since aOR of having a pathological salivary flow (SF < 0.1 mL/min) was 1.23 CI 95% [0.49–2.64] (*p* = 0.58), 0.71 CI 95% [0.24–1.43] (*p* = 0.38) and 0.83 CI 95% [0.34–1.68] (*p* = 0.6), in spring, summer and winter, respectively, in comparison to values collected in fall (Additional file 1: Table S2).

The influence on PROs of age, symptomatic treatments of dryness and immunomodulatory treatments was also analysed in the ASSESS cohort. All the variations of pain, fatigue and dryness VASs and of the ESSPRI score over seasons, adjusted on age and treatment regimen were very limited and did not reach statistical significance. Adjusted pain VASs variations were 1.3 on a 100-unit scale, CI 95% [− 1.6; 4.4] (*p* = 0.4), 0/100 CI 95% [− 3; 2.9] (*p* = 0.99) and − 0.3/100 CI 95% [− 3; 2.4] (*p* = 0.83), in spring, summer and winter, respectively, in comparison to values collected in fall. Adjusted fatigue VASs variations were 1.3/100 CI 95% [− 1.3; 4] (*p* = 0.33), 0.4/100 [− 2.2; 3] (*p* = 0.79), and − 0.2/100 [− 2.7; 2.2] (*p* = 0.84) in spring, summer and winter, respectively, in comparison to values collected in fall. Adjusted dryness VASs variations were 1.3/100 CI 95% [− 1; 3.6] (*p* = 0.27), 2/100 CI 95% [− 0.3; 4.3] (*p* = 0.09) and 0.6/100 CI 95% [− 1.5; 2.8] (*p* = 0.55), respectively, in comparison to values collected in fall. Finally, adjusted ESSPRI score changes were of 1.2/100 CI 95% [− 0.8; 3.2] (*p* = 0.25), 0.6/100 CI 95% [− 1.4; 2.6] (*p* = 0.55) and − 0.2/100 CI 95% [− 2.1; 1.6] (*p* = 0.82), respectively, in comparison to values collected in fall (Additional file 1: Table S3).

## Discussion

The present study performed on a large population of patients and on a long duration did not show any seasonal effect on the main symptoms of pSS, pain, fatigue and dryness.

The pathogenesis of pSS is, not unlike other auto-immune diseases, considered multifactorial. A complex relationship between environmental and immunological factors affecting a peculiar genetic background may interact and sustain disease onset in susceptible individuals.

A seasonal pattern of several suspected environmental triggers, such as viral infections (EBV and CMV in winter and HBV and HCV in spring and summer) as well as sunshine exposure (through UV-B radiation, governing blood levels of inactive 25OH-vitamin D_3_), might suggest an association between disease activity and season changes. Of note, previous studies have postulated that low levels of vitamin D, because of its immunomodulatory effects, could affect clinical manifestations in patients with pSS [17], as demonstrated in other IMIDs, especially in LES [18] and RA [19]. Nevertheless, vitamin D influence on pSS activity remains controversial.

Otherwise, weather-related ocular and oral dryness enhancement has been reported in patients outside autoimmune context, mainly in spring and summer, but this has never been specifically analysed in pSS.

Indeed, to our knowledge, there is no study available in the literature investigating a seasonal impact on symptoms and outcomes in primary Sjögren’s syndrome.

In the present study, all the fluctuations observed were not statistically significant and were not clinically relevant either, since they were well lower than the minimal clinically important improvements (MCIIs) for dryness, pain and fatigue, which are − 10, − 10 and − 20 on a 100-point scale, respectively [15].

Nevertheless, this study has several limitations. First, patients included in the ASSESS cohort underwent an annual evaluation of their symptoms and activity, mostly at the same period every year. However, the three trials lasted 1 year and visits were scattered all over the year. In addition, the statistical analysis not only focused on the intra-individual but also assessed the inter-individual seasonal variability of symptoms.

Several confounding factors, such as age, symptomatic treatments of dryness and immunomodulatory treatments assumed by patients, could have hampered the interpretation of the results. To address this point, linear mixed models were adjusted to take into account the influence of age and treatments on objective assessment of ocular and oral dryness and on PROs, in the ASSESS cohort. Adjusted analyses on age, immunomodulatory drug exposure and symptomatic treatments of dryness did not reveal any statistically significant effect of seasons on objective dryness outcomes. Furthermore, even when adjusted on age and treatments, there was no significant effect of seasons on fatigue, pain and dryness VASs or on the ESSPRI score either.

Other limitations include the unavailability of weather variables such as temperature, relative humidity, barometric pressure, sunshine exposure, precipitations and the variability of geographic locations (multicentric studies). A longitudinal study involving meteorological features and assessing pSS outcomes every months during several years, as recently performed in RA [20], could be the most accurate way to investigate the effect of seasonality in pSS.

## Conclusions

This first large study on seasonality in pSS provides new evidence that fatigue, pain and dryness, as well as the ESSPRI score, do not have meaningful fluctuations according to months or seasons. In pSS, seasonality does not affect patient-reported outcomes (PROs) on fatigue, pain and dryness.

## Supplementary information

**Additional file 1:** **Table S1.** Drug regimens used at enrolment in the ASSESS cohort and number of patients with systemic immuno-modulatory drugs and symptomatic treatments of dryness. **Table S2.** Influence of seasons on objective assessments of ocular and oral dryness (Schirmer test ≤5 mm; Salivary flow ≤0.1 mL/min). **Table S3.** Variations of pain, fatigue and dryness VASs (on a 100-unit scale) and of the ESSPRI score according to seasons, adjusted on age, immunosuppressive treatments and symptomatic treatments of dryness.

## Data Availability

The datasets used and/or analysed during the current study are available from the corresponding author on reasonable request.

## Abbreviations

ASSESS: Assessment of Systemic Signs and Evolution in Sjögren’s Syndrome
CI: Confidence interval
CMV: Cytomegalovirus
CYC: Cyclophosphamide
EBV: Epstein-Barr virus
ESSPRI: EULAR Sjögren’s Syndrome Patient Reported Index
EULAR: European League Against Rheumatism
HBV: Hepatitis B virus
HCV: Hepatitis C virus
IMID: Immune-mediated inflammatory disease
IS: Immunosuppressive drugs
MCII: Minimal clinically important improvement
aOR: Adjusted odds ratio
PROs: Patient-reported outcomes
pSS: Primary Sjögren’s Syndrome
RA: Rheumatoid arthritis
RCT: Randomised controlled trial
RTX: Rituximab
SF: Unstimulated salivary flow
SLE: Systemic lupus erythematosus
VAS: Visual analogue scale
