# Supplementary material for: Seasonal effect on fatigue, pain and dryness in primary Sjögren’s syndrome
Source: Arthritis Res Ther. 2020 Feb 24;22:39. doi: 10.1186/s13075-020-2118-1 (PMC7041128; doi:10.1186/s13075-020-2118-1)
Supplement: Supplementary file 1 — Additional file 1: Table S1. Drug regimens used at enrolment in the ASSESS cohort and number of patients with systemic immuno-modulatory drugs and symptomatic treatments of dryness. Table S2. Influence of seasons on objective assessments of ocular and oral dryness (Schirmer test ≤5 mm; Salivary flow ≤0.1 mL/min). Table S3. Variations of pain, fatigue and dryness VASs (on a 100-unit scale) and of the ESSPRI score according to seasons, adjusted on age, immunosuppressive treatments and symptomatic treatments of dryness. [file 13075_2020_2118_MOESM1_ESM.docx]

**Table S1** Drug regimens used at enrolment in the ASSESS cohort and number of patients with systemic immuno-modulatory drugs and symptomatic treatments of dryness.

| **Treatment modality** | **n (%)** |
| --- | --- |
| **Symptomatic treatments of dryness** |  |
| **Topical eye drops** | 268 (67.8) |
| **Pilocarpine hydrochloride** | 100 (25.3) |
|  |  |
| **Systemic immunomodulatory treatments** |  |
| **Corticosteroids** | 95 (24.1) |
| **Hydroxychloroquine** | 121 (30.6) |
| **Methotrexate** | 20 (5.1) |
| **Leflunomide** | 2 (0.5) |
| **Azathioprine** | 6 (1.5) |
| **Mycophenolate mofetil** | 5 (1.3) |
| **Cyclophosphamide** | 3 (0.8) |
| **Rituximab** | 4 (1) |
| **≥1 IS*** | 37 (9.4) |

*IS=Immunosuppressive drugs;

**Table S2** Influence of seasons on objective assessments of ocular and oral dryness (Schirmer test ≤ 5 mm; Salivary flow ≤ 0.1mL/min).

| **Season** | **Eye dryness ORa*[95%CI]** | **P value** | **Oral dryness ORa* [95% CI]** | **P value** |
| --- | --- | --- | --- | --- |
| **Fall^#^** | 1 | - | 1 | - |
| **Summer** | 0.95 [0.43-2.14] | 0.89 | 0.71 [0.24-1.43] | 0.38 |
| **Winter** | 0.75 [0.38-1.53] | 0.35 | 0,83 [0.34-1.68] | 0.6 |
| **Spring** | 0.93 [0.42-1.8] | 0.82 | 1,23 [0,5-2.64] | 0.58 |

*Odds ratios were adjusted on age, immunosuppressive treatment regimens and symptomatic treatments of dryness. ^#^ Oral and ocular dryness values collected in fall were defined as reference for these analyses.

**Table S3** Variations of pain, fatigue and dryness VASs (on a 100-unit scale) and of the ESSPRI score according to seasons, adjusted on age, immunosuppressive treatments and symptomatic treatments of dryness.

| **Differences between seasons*** | **Spring** | **P value** | **Summer** | **P value** | **Fall^#^** | **Winter** | **P value** |
| --- | --- | --- | --- | --- | --- | --- | --- |
| **Pain [95% CI]** | 1.3 [-1.6; 4.4] | 0.4 | 0 [-3; 2.9] | 0.99 | _ | -0.3 [-3; 2.4] | 0.83 |
| **Fatigue [95% CI]** | 1.3 [-1.3; 4] | 0.33 | 0.4 [-2.2; 3] | 0.79 | _ | -0.2 [-2.7; 2.2] | 0.84 |
| **Dryness [95% CI]** | 1.3 [-1; 3.6] | 0.27 | 2 [-0.3; 4.3] | 0.09 | _ | 0.6 [-1.5; 2.8] | 0.55 |
| **ESSPRI [95% CI]** | 1.2 [-0.8; 3.2] | 0.25 | 0.6 [-1.4; 2.6] | 0.55 | _ | -0.2 [-2.1; 1.6] | 0.82 |

*Improvements (in mm) of VASs and of the ESSPRI score between seasons, adjusted on age, immuno-suppressive treatment regimens and symptomatic treatments of dryness. ^#^ Values collected in fall were defined as reference for these analyses.
